# Transarterial infusion chemotherapy with cisplatin plus S-1 for hepatocellular carcinoma treatment: a phase I trial

**DOI:** 10.1186/1471-2407-14-301

**Published:** 2014-04-30

**Authors:** Tetsuji Terazawa, Shunsuke Kondo, Hiroko Hosoi, Chigusa Morizane, Satoshi Shimizu, Shuichi Mitsunaga, Masafumi Ikeda, Hideki Ueno, Takuji Okusaka

**Affiliations:** 1 Department of Hepatobiliary and Pancreatic Oncology, National Cancer Center Hospital, Tokyo, Japan; 2 Department of Experimental Therapeutics, Exploratory Oncology Research & Clinical Trial Center, National Cancer Center, 5-1-1 Tsukiji Chuo-ku, 104-0045 Tokyo, Japan; 3 Department of Hepatobiliary and Pancreatic Oncology, National Cancer Center Hospital East, Chiba, Japan

**Keywords:** Hepatocellular carcinoma, Transarterial infusion chemotherapy, Cisplatin, S-1

## Abstract

**Background:**

In Japan, transarterial infusion chemotherapy using cisplatin (CDDP-TAI) is frequently used for advanced hepatocellular carcinoma (HCC). Moreover, oral chemotherapy with S-1, an oral fluoropyrimidine derivative, has also elicited promising responses in HCC patients. We determined the recommended dosage for CDDP-TAI plus S-1 combination therapy for advanced HCC.

**Methods:**

Twelve Child–Pugh class A or B patients with advanced HCC who met the eligibility criteria were enrolled in this phase I trial. Patients received CDDP-TAI (infusion, day 1) plus S-1 (oral administration, days 1–21) every 5 weeks until disease progression.

**Results:**

Cisplatin (65 mg/m^2^) was administered with S-1 at 50 mg · m^-2^ day^-1^ (level 1, 3 patients), 60 mg · m^-2^ day^-1^ (level 2, 3 patients), or 80 mg · m^-2^ day^-1^ (level 3, 6 patients). The total number of treatment courses was 25 (median, 2 courses/patient; range, 1–6 courses). Dose-limiting toxicity was not observed in any patient at any level; therefore, the recommended dosage for cisplatin and S-1 in combination was level 3. Grade 3 adverse events were elevated alanine aminotransferase levels (2 patients), elevated aspartate aminotransferase levels (2 patients), anemia (1 patient), and decreased platelet counts (1 patient). Median progression-free survival and overall survival were 73 days and 328 days, respectively. The disease control rate was 58% (7/12); 17% (2/12) of patients achieved partial response and 42% (5/12) achieved stable disease. CDDP-TAI plus S-1 is safe for the treatment of HCC.

**Conclusion:**

The recommended dosage for further evaluation of this combination therapy in phase II studies is 65 mg/m^2^ CDDP and 80 mg/m^2^ S-1.

**Trial registration:**

UMIN; number: UMIN000003113

## Background

Hepatocellular carcinoma (HCC) is one of the most common cancers worldwide. Surgical resection, liver transplant, percutaneous ethanol injection (PEI), and radiofrequency ablation (RFA) are considered the mainstay of treatment for patients with potentially curable disease. Transcatheter arterial chemoembolization (TACE) is the treatment of choice for non-curative HCC. HCC patients with advanced stage disease or disease progression after locoregional therapy have a dismal prognosis owing to few effective treatment options and rapid tumor progression [1]. Recently, several placebo-controlled, randomized phase III trials have shown that sorafenib, an oral multi-tyrosine kinase inhibitor, provided a significant survival benefit in patients with advanced HCC [2,3]. However, sorafenib only increased median survival by approximately 3 months when compared with placebo. Therefore, further development of effective therapeutic strategies for the treatment of advanced HCC is essential.

S-1, an oral fluoropyrimidine derivative, is clinically effective against various solid tumors and has an acceptable toxicity profile. The response rate of S-1 against HCC was reported to be 21.3% in a phase II study [4]. Hepatic transcatheter arterial infusion chemotherapy using cisplatin (CDDP-TAI) has also shown a favorable tumor response rate of 33.8% in advanced HCC patients in a phase II clinical study [5]. The combination of CDDP with the fluoropyrimidine 5-fluorouracil (5-FU) has been shown to exhibit significant synergy against various cancer types *in vitro* and *in vivo*[6,7]. Given the antitumor activity of S-1 and CDDP against HCC, combination therapy may improve patient outcome. Therefore, we conducted a phase I clinical trial of CDDP-TAI plus S-1 in patients with advanced HCC. The primary endpoint was to determine the recommended dosage (RD) of CDDP-TAI plus S-1 for phase II studies based on the maximum-tolerated dose (MTD) and dose-limiting toxicity (DLT). The secondary endpoints were to evaluate toxicity, progression-free survival (PFS), and overall survival (OS).

## Methods

This study was approved by the ethics committee of National Cancer Center Hospital. The trial was designed according to the current Declaration of Helsinki (Somerset West, South Africa, 1996). Written informed consent was obtained from each participating patient before enrollment. This study was registered with the University Hospital Medical Information Network in Japan (UMIN; number UMIN000003113) and has been completed.

### Patient eligibility

We used the following eligibility criteria to screen patients for inclusion: histological or clinical confirmation of HCC by early tumor staining with dynamic computed tomography or dynamic magnetic resonance imaging; elevated serum levels of alpha-fetoprotein (AFP) or protein induced by vitamin K absence-II (PIVKA-II); age ≥ 20 years; unresectable HCC; no indication for liver transplantation, local ablation therapy (percutaneous RFA, PEI, and microwave coagulation), and TACE; Child–Pugh class A or B; Eastern Cooperative Oncology Group performance status (PS) 0–2; adequate bone marrow, renal, and cardiac function; existence of a intrahepatic lesion; adequate oral intake; and life expectancy > 60 days. For eligibility, patients also had to meet the following clinical laboratory test criteria: neutrophil count ≥ 1,500/mm^3^; platelet count ≥ 60,000/mm^3^; hemoglobin (Hb) ≥ 9.0 g/dL; serum creatinine (Cr) ≤ 1.2 mg/dL and Cr clearance ≥ 50 mL/min; alanine aminotransferase (ALT) ≤ 5 × the upper limit of the normal range (ULN); aspartate aminotransferase (AST) ≤ 5 × ULN; serum albumin ≥ 2.8 g/dL; prothrombin time and international normalized ratio ≤ 2.3; and serum total bilirubin level (T-Bil) ≤ 2.0 mg/dL.

The exclusion criteria were as follows: any treatment for HCC within 28 days before study entry; prior chemotherapy with 5-FU or a platinum-containing drug; administration of blood transfusion, blood preparation, albumin preparation, or granulocyte-colony stimulating factor within 15 days before study entry; regular use of phenytoin, warfarin, or flucytosine; severe heart failure; uncontrollable diabetes mellitus; active infection; pregnancy or lactation; childbearing age for women unless effective contraception was being used; severe drug hypersensitivity; mental disorder; watery diarrhea; moderate or marked pleural effusion or ascites; and other serious medical conditions.

### Treatment

**Figure 1:**
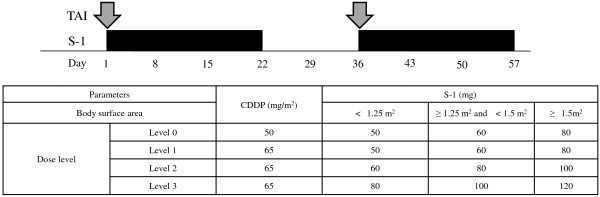
Treatment schedule.

Patients received CDDP-TAI (infusion on day 1) and S-1 (daily oral administration on days 1–21) every 5 weeks. CDDP-TAI was performed via a lobar or selective approach depending on tumor number and location Figure 1. Treatment was continued until occurrence of disease progression or unacceptable adverse events. If patients did not fulfill the continuation criteria (absolute white blood cell count ≥ 2000/mm^3^; platelet count ≥ 50,000/mm^3^; Cr ≤ 1.5 mg/dL; diarrhea or mucositis ≤ grade 2; and rash ≤ grade 2), S-1 was temporarily suspended until recovery. The next course was started only when patients fulfilled the following criteria: absolute neutrophil count ≥ 1000/mm^3^; Hb ≥ 9.0 g/dL; platelet count ≥ 50,000/mm^3^; AST ≤ grade 2; ALT ≤ grade 2; Cr ≤ 1.2 mg/dL; diarrhea or mucositis ≤ grade 2; and rash ≤ grade 2. Patients who required > 49 days to begin the next cycle were withdrawn from the study.

Patients were scheduled to receive CDDP-TAI plus S-1 at 3 dosage levels (Table 1). CDDP was administered at a dosage of 65 mg/m^2^ at levels 1–3. S-1 was administered as follows: level 1, 50 mg/m^2^; level 2, 60 mg/m^2^; and level 3, 80 mg/m^2^. At level 0, the CDDP dosage was decreased to 50 mg/m^2^, and S-1 was administered at the same dosage as level 1.

**Table 1:** Dose escalation

		**CDDP (mg/m**^**2**^**)**	**S-1 (mg)**		
			**BSA < 1.25 m**^**2**^	**1.5 m**^**2**^ **> BSA ≥ 1.25 m**^**2**^	**BSA ≥ 1.5 m**^**2**^
Dose level	Level 0	50	50	60	80
	Level 1*	65	50	60	80
	Level 2	65	60	80	100
	Level 3	65	80	100	120

CDDP, cisplatin; BSA, body surface area; *, starting dose.

### Study design

The objective of this trial was to evaluate the frequency of DLT during the first 28 days of cycle 1 and to determine the RD that should be used in a phase II trial. At least 3 patients were enrolled at each dosage level. Level 1, which was the starting dose, was initially administered to 3 patients. If DLT was observed in 1 or 2 of the initial 3 patients, up to 3 additional patients were enrolled at the same dosage level. The highest dosage level that did not cause DLT in 3 of 3 patients or ≥ 3 of 6 patients treated during the first cycle was considered the MTD. DLT was defined as febrile neutropenia; clinically or microbiologically documented infection with grade 3 or 4 neutrophils; absolute grade 4 leukopenia or neutropenia for ≥ 7 days; grade 4 thrombocytopenia or administration of platelet transfusion; grade 3 non-hematological toxicity; AST, ALT, ALP or γ-glutamyltranspeptidase >15 × ULN; Cr > 2.0 mg/dL; any toxicity that required interruption of therapy for > 49 days between day 1 of the first cycle and day 1 of the second cycle; and interruption of S-1 chemotherapy more than 21 times during 1 cycle. Toxicity was graded according to the Common Terminology Criteria for Adverse Events version 3.0.

### Assessment of efficacy and toxicity

All patients who received at least 1 dose of the study drugs were included in the response and toxicity evaluations. Every 5 weeks, tumor responses were assessed according to the Response Evaluation Criteria in Solid Tumors version 1.0. PFS was defined as the interval between the date of treatment initiation and the date of first confirmed disease progression or the date of death from any cause. OS was defined as the interval from the date of treatment initiation to the date of death from any cause. Median PFS and median OS were estimated using the Kaplan–Meier method. For each cycle, tumor markers (AFP and PIVKA-II) were assessed 5 weeks after CDDP-TAI administration.

## Results

### Patient characteristics and treatment

Between January 2010 and June 2011, 13 patients were enrolled at the National Cancer Center Hospital and National Cancer Center Hospital East in Japan. Of the 13 enrolled patients, 12 patients were eligible for the toxicity and efficacy evaluations (level 1, 3 patients; level 2, 3 patients; and level 3, 6 patients). One patient was excluded before initiation of the protocol treatment because of the use of a drug that had interactions with S-1. Patient characteristics are shown in Table 2. Eleven (91.7%) patients were PS 0, and 1 (8.3%) patient was PS 1. Seven (58.3%) patients were Child–Pugh score 5, 3 (25%) patients were Child–Pugh score 6, and 2 (16.7%) patients were Child-Pugh score 7 at baseline. Four (33%) patients had metastatic disease. Metastatic organs were the lung (4 patients), bone (1 patient), and lymph node (1 patient). Five (41.7%) patients had received prior treatment with sorafenib. Eleven (91.7%) patients had a history of TACE. Epirubicin was used for prior TACE. There was no history of chemotherapy, except for sorafenib. The total number of treatment courses was 25, with a median of 2 courses per patient (range, 1–6 courses).

**Table 2:** Patient characteristics

		**Level 1 (n = 3) n (%)**	**Level 2 (n = 3) n (%)**	**Level 3 (n = 6) n (%)**	**Total (n = 12) n (%)**
Age	Median	62	67	64.5	64.5
	Range	47–68	62–79	47–80	47–80
PS	0	3	3	5	11 (91.7)
	1	0	0	1	1 (8.3)
	2	0	0	0	0 (0)
TNM staging	Stage II	1	1	1	3 (25.0)
	Stage IIIA	0	0	3	3 (25.0)
	Stage IIIB	1	1	0	2 (16.7)
	Stage IVA	0	0	0	0 (0)
	Stage IVB	1	1	2	4 (33.3)
Virus infection	HBV	2	2	3	7 (58.3)
	HCV	1	1	1	3 (25.0)
	Non-B, non-C	0	0	2	2 (16.7)
Child–Pugh score	5	3	2	2	7 (58.3)
	6	0	1	2	3 (25.0)
	7	0	0	2	2 (16.7)
Extrahepatic metastasis	No	2	2	4	8 (66.7)
	Yes	1	1	2	4 (33.3)
History of sorafenib	No	2	2	3	7 (58)
	Yes	1	1	2	5 (42)
History of TACE	No	0	0	1	1 (8.3)
	Yes	3	3	5	11 (91.7)

PS, Eastern Cooperative Oncology Group performance status; TNM, tumor node metastasis according to American Joint Committee on Cancer; HBV, hepatitis B virus; HCV, hepatitis C virus; TACE, transcatheter arterial chemoembolization.

### Toxicity

Treatment was generally well tolerated throughout the study, and grade 4 toxicity was not observed in any of the patients (Table 3). Grade 3 toxicity occurred in 5 of 12 (41.7%) patients. The grade 3 adverse events were elevated AST (17%), elevated ALT (17%), anemia (17%), thrombocytopenia (8%), elevated T-Bil (8%), hyponatremia (8%), and hemorrhage in the biliary tree (8%). Grade 3 elevated AST, elevated ALT, anemia, and thrombocytopenia recovered without any specific treatments. Grade 3 hyponatremia was reversible with conservative treatment. Moreover, in a patient with bile duct invasion of the tumor, grade 3 elevated T-Bil and hemorrhage in the biliary system due to tumor invasion disappeared after transcatheter arterial embolization. On the other hand, DLT was not observed during the first cycle at any dosage level; therefore, the RD was determined to be level 3.

**Table 3:** Adverse events in all cycles

	**Level 1 (n = 3)**		**Level 2 (n = 3)**		**Level 3 (n = 6)**		**Total (n = 12)**	
	**All G n (%)**	**G3–4 n (%)**	**All G n (%)**	**G3–4 n (%)**	**All G n (%)**	**G3–4 n (%)**	**All G n (%)**	**G3–4 n (%)**
Leukopenia	3 (100)	0 (0)	3 (100)	0 (0)	3 (50)	0 (0)	9 (75)	0 (0)
Neutropenia	2 (67)	0 (0)	3 (100)	0 (0)	2 (33)	0 (0)	7 (58)	0 (0)
Anemia	3 (100)	0 (0)	2 (67)	1 (33)	2 (33)	1 (17)	7 (58)	2 (17)
Thrombocytopenia	3 (100)	0 (0)	3 (100)	1 (33)	3 (50)	0 (0)	9 (75)	1 (8)
Total bilirubin	2 (67)	0 (0)	2 (67)	1 (33)	6 (100)	0 (0)	10 (83)	1 (8)
AST	2 (67)	0 (0)	3 (100)	2 (67)	3 (50)	0 (0)	8 (67)	2 (17)
ALT	3 (100)	1 (33)	2 (67)	1 (33)	1 (17)	0 (0)	6 (50)	2 (17)
ALP	2 (67)	0 (0)	0 (0)	0 (0)	1 (17)	0 (0)	3 (25)	0 (0)
Hyponatremia	2 (67)	0 (0)	1 (33)	0 (0)	5 (83)	1 (17)	8 (67)	1 (8)
Hypoalbuminemia	2 (67)	0 (0)	1 (33)	0 (0)	3 (50)	0 (0)	6 (50)	0 (0)
Fatigue	2 (67)	0 (0)	2 (67)	0 (0)	4 (67)	0 (0)	8 (67)	0 (0)
Anorexia	1 (33)	0 (0)	3 (100)	0 (0)	4 (67)	0 (0)	8 (67)	0 (0)
Nausea	3 (100)	0 (0)	2(67)	0 (0)	1 (17)	0 (0)	6 (50)	0 (0)
Vomiting	0 (0)	0 (0)	1 (33)	0 (0)	0 (0)	0 (0)	1 (8)	0 (0)
Diarrhea	0 (0)	0 (0)	1 (33)	0 (0)	1 (17)	0 (0)	2 (17)	0 (0)
Stomatitis	2 (67)	0 (0)	1 (33)	0 (0)	1 (17)	0 (0)	4 (33)	0 (0)
Edema	1 (33)	0 (0)	0 (0)	0 (0)	3 (50)	0 (0)	4 (33)	0 (0)
Hemorrhage in biliary tree	0 (0)	0 (0)	1 (33)	1 (33)	0 (0)	0 (0)	1 (8)	1 (8)

G, grade; AST, aspartate aminotransferase; ALT, alanine aminotransferase; ALP, alkaline phosphatase.

### Efficacy

**Figure 2:**
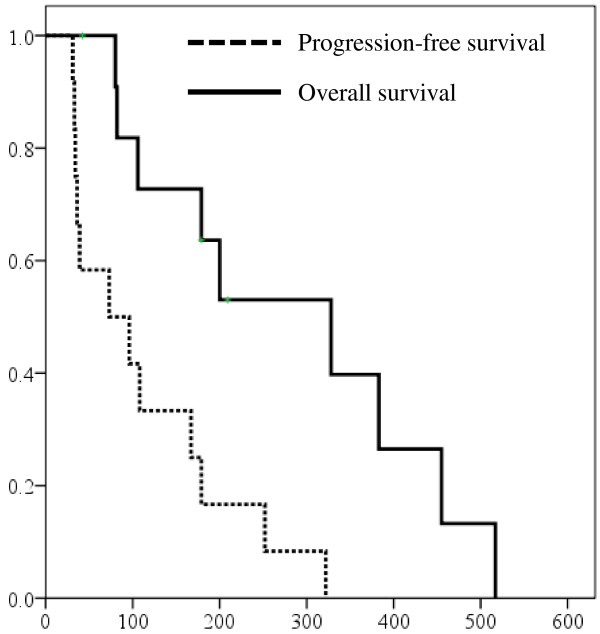
Progression-free survival (PFS) and overall survival (OS) (n = 12). The median PFS and OS were 73 days and 328 days, respectively.

The PFS and OS for all 12 patients in this phase I study are shown in Figure 2. The median PFS and OS were 73 days (95% confidence interval [CI], 0–169) and 328 days (95% CI, 128–527), respectively. The response rate was 17%. The disease control rate (partial response [PR] + stable disease [SD]) was 58% (PR, 2 patients; SD, 5 patients; and progressive disease [PD], 5 patients). One patient had PR and 2 patients had SD at level 1; 1 patient had PR and 2 patients had PD at level 2; and 3 patients had SD and 3 patients had PD at level 3. Five patients who had received prior treatment with sorafenib had a response rate of 40%, disease-control rate of 80% (PD, 1 patient; SD, 2 patients; PR, 2 patients), and median PFS of 179 days (95% CI, 26.6–331.4).

## Discussion

This is the first study to estimate the safety and efficacy of CDDP-TAI plus S-1 combination therapy in advanced HCC patients. This study was conducted to determine the RD of this combination therapy for subsequent phase II studies. DLT was not observed during the first cycle at any dose level; therefore, the RD was determined to be 65 mg/m^2^ CDDP and 80 mg/m^2^ S-1. Grade 3 adverse events in all cycles included elevated AST, elevated ALT, anemia, and thrombocytopenia. CDDP-TAI plus S-1 combination therapy showed an acceptable toxicity profile.

In our study, the most frequent grade 3 adverse event was elevated AST and ALT levels, which are indicative of liver injury. CDDP-TAI plus S-1 induced more severe hepatic adverse events in advanced HCC patients than a comparable dosage of S-1 plus intravenous CDDP in gastric cancer patients [8]. The incidence of grade 3 or 4 elevated AST and ALT in advanced HCC patients was similar between CDDP-TAI plus S-1 in our study and CDDP-TAI alone in a previous study (AST, 32.5%; ALT, 11.3%) [5]. Therefore, addition of S-1 to CDDP-TAI may not affect liver function, and CDDP-TAI itself may be responsible for hepatic toxicity. Liver injury and cirrhosis reduce the ability of the liver to metabolize and excrete drugs, which may further exacerbate liver damage. In the present study, 8 of 12 patients were infected with hepatitis B or C virus. Thus, CDDP-TAI also appeared to have a negative effect on non-cancerous liver tissue. However, CDDP-TAI toxicity was managed without any specific treatments.

This study showed that the standard S-1 dosage was well tolerated by HCC patients with Child–Pugh score 5–7 who received CDDP-TAI. Severe diarrhea and stomatitis, which are often associated with S-1 toxicity [9], were not reported. Moreover, grade 3 or 4 hepatic toxicity did not occur in any of the patients at the highest S-1 dosage (level 3). The liver plays an important role in the metabolism of tegafur, which is the main component of S-1. The conversion of tegafur to 5-FU is mainly mediated by hepatic cytochrome CYP2A6 [10]. 5-FU is rapidly metabolized by dihydropyrimidine dehydrogenase in the liver after intravenous administration of 5-FU alone. However, Furuse et al. reported that hepatic dysfunction associated with Child–Pugh B did not affect the pharmacokinetics of S-1 (80 mg/m^2^) or 5-FU [4]. This study also showed that the standard dose of S-1, even when used in combination with CDDP-TAI, was tolerable in patients with mild liver injury. As a limitation, although this study included 2 patients with a Child-Pugh score of 7 at level 3, no patients with a Child-Pugh score of 8–9 were included. This is because the present trial was a phase I study, and therefore, only 12 patients were included, who tended to have a good score. We will plan a phase II study with a larger number of patients with HCC to evaluate the efficacy and toxicity.

CDDP-TAI plus S-1 combination therapy affords a certain level of tumor control for patients with advanced HCC. Moreover, CDDP-TAI plus S-1 combination therapy was effective as second-line treatment in advanced HCC patients who had received prior sorafenib; 40% of patients achieved a partial response and 40% of patients achieved stable disease. A double-blind, placebo-controlled phase III trial to evaluate the efficacy of S-1 in patients with advanced HCC failure to sorafenib monotherapy is currently ongoing (JapicCTI-090920). In the future, we plan to validate the efficacy of CDDP-TAI plus S-1 combination therapy against advanced HCC that has progressed after sorafenib in a phase II study.

## Conclusions

This study showed that CDDP-TAI plus S-1 can be safely used in the treatment of advanced HCC. The RD for a subsequent phase II clinical trial was estimated to be 65 mg/m^2^ CDDP and 80 mg/m^2^ S-1. To assess the efficacy of this combination therapy against advanced HCC, we plan to conduct a subsequent phase II study.

## Competing interests

The authors declare that they have no competing interests.

## Authors’ contributions

SK, CM, HU, and TO designed and contributed to all stages of the study. TT, SK, and HH participated in statistical analyses and discussion of the results. SK, CM, SS, SM, MI, HU, and TO recruited the patients. TT and HH helped in drafting the manuscript. All the authors read and approved the final manuscript.

## Pre-publication history

The pre-publication history for this paper can be accessed here:

http://www.biomedcentral.com/1471-2407/14/301/prepub
